# Impact of the Social Vulnerability Index on Pulmonary Embolism Mortality

**DOI:** 10.1016/j.chpulm.2023.100006

**Published:** 2023-06-16

**Authors:** Ramzi Ibrahim, Rebecca Wig, Roger Y. Kim, Bryan S. Benn, See-Wei Low

**Affiliations:** Department of Medicine (C. R.-G. and L. V. C.), University of Arizona—Banner University Medical Center; the University of Arizona College of Medicine (C. V. G. T.); the Department of Medicine (R. Y. K.), Division of Pulmonary, Allergy, and Critical Care, University of Pennsylvania; and the Division of Pulmonary Medicine (B. S. B. and S.-W. L.), Respiratory Institute, Cleveland Clinic.

## To the Editor:

Pulmonary embolism (PE) is a significant cause of morbidity and mortality influenced by social determinants of health. The Social Vulnerability Index (SVI) measures social determinants of health in US counties based on 16 social attributes and has been used to study the impact of social vulnerability on multiple health outcomes, including all-cause and cardiovascular mortality and disability (Table 1).^1–3^ We used the SVI to investigate patterns of PE-related mortality in all US counties from 2014 to 2018.

We obtained county-level mortality and SVI data from the CDC Wide-Ranging Online Data for Epidemiologic Research (WONDER) and Agency for Toxic Substances and Disease Registry databases, respectively.^1,4^ Mortality data were based on death certificate information, including underlying causes of death and demographic information. Confidentiality constraints led to suppression of mortality data when there were subnational death counts. All diagnoses were based on the *International Classification of Diseases, Tenth Revision*.

All mortality data related to PE (*International Classification of Diseases, Tenth Revision:* I26) from 2014 to 2018 were queried. SVI values from 2014 to 2018 were assigned percentiles from 0 to 1, with higher values indicating greater social vulnerability. SVI percentile rankings were then divided into four quartiles (SVI-Q) based on overall rankings (0–0.25 as 1st quartile [SVI-Q1] and least vulnerable, and > 0.75–1.00 as SVI-Q4 and most vulnerable), similarly done by Khan et al.^3^

Crude mortality rates, population sizes, and CIs can be found in the CDC WONDER database.^4^ Age-adjusted mortality rates (AAMR) per 100,000 population and CIs were estimated for the overall population and stratified by demographic variables and SVI quartiles. A negative impact by SVI was characterized by a higher AAMR within SVI-Q4 compared with SVI-Q1 and nonoverlapping CIs, except for American Indian/Alaska Native individuals, because AAMR in SVI-Q1 was under confidentiality constraints. Rate differences were calculated as excess or fewer deaths per 100,000 person-years. Because the data are publicly available and de-identified, institutional review board approval was not necessary.

Between 2014 and 2018, 42,850 PE-related deaths occurred, with a consistent AAMR of 2.27 in 2014 to 2.30 in 2018. SVI-Q4 had a higher AAMR (2.43) and accounted for 0.46 excess deaths per 100,000 person-years compared with SVI-Q1 (1.97) (Table 2). Male individuals (2.39) had a higher AAMR than female individuals (2.23), and both sexes were negatively impacted by higher social vulnerability (0.57 and 0.43 excess deaths per 100,000 person-years, respectively).

The highest AAMR was among those aged ≥ 65 years (10.90), followed by ages 35 to 64 years (2.16) and < 35 years (0.22). All age groups were negatively impacted by higher social vulnerability (1.28, 0.70, and 0.05 excess deaths per 100,000 person-years, respectively).

Non-Hispanic populations (2.47) had a higher AAMR and were negatively impacted by the SVI (0.87 excess deaths per 100,000 person-years), unlike Hispanic populations (AAMR 0.98). Black populations had the highest AAMR (4.10), followed by White (2.19), American Indian/Alaska Native (1.18), and Asian/Pacific Islander populations (0.46). Black and White populations were negatively impacted by higher SVI (0.95 and 0.23 excess deaths per 100,000 person-years, respectively), whereas the AAMR among American Indian/Alaska Native and Asian/Pacific Islander populations was not negatively impacted by the SVI.

The South had the highest AAMR (2.88), followed by the Midwest (2.60), Northeast (1.81), and West (1.52). SVI had a negative impact on the midwestern and southern regions (1.24 excess deaths per 100,000 person-years each), but not on western and northeastern regions. Nonmetropolitan regions (3.41) had a higher AAMR than metropolitan regions (2.12) and were negatively impacted by the SVI (1.9 excess deaths per 100,000 person-years), whereas metropolitan regions were not negatively impacted.

Our analysis identified a consistent mortality rate related to PE from 2014 to 2018, with significant disparities among sex, racial, and geographic populations. Most included populations were negatively impacted by increasing social vulnerability.

Although both sexes are negatively affected by increasing social vulnerability, our analysis revealed a sex disparity in PE mortality. Female individuals have a higher prevalence of risk factors for PE, but no sex-based differences in PE severity or outcomes have been observed.^5^ These conflicting findings emphasize the importance of continued investigation to better inform optimal care and mitigate disparities. In congruence with our findings, racial differences in the received care and outcomes of patients with PE have been reported.^6^ Black individuals are more likely to present with severe PE; to experience earlier onset and long-term disability; have higher odds of PE related mortality; and have lower rates of intervention, including catheter-based and surgical interventions such as suction thrombectomy, catheter-directed thrombolysis, and surgical embolectomy.^6,7^ This racial disparity may be explained by accessibility to care and differences in availability of resources, structural racism, and implicit biases.^6,8^

The SVI strongly correlated with higher mortality among many of our included populations. The SVI has been associated with high rates of diabetes, chronic kidney disease, hypertension, and atherosclerotic disease, likely contributing to the increased risk of PE mortality.^9,10^

Our study has limitations. The use of death certificates to capture all PE deaths is subject to potential inaccuracies and undersampling. However, these inaccuracies are unlikely to explain the observed disparities alone. Reverse association is also a possibility because increased mortality related to PE also may increase social vulnerability. Furthermore, given the cross-sectional design of our study, causality cannot be established. Finally, we did not adjust for other risk factors associated with increasing PE mortality in our analyses. Our study is strengthened by its use of a widely available database that captures all US mortality data through the National Center for Health Statistics.

Our findings suggest potential disparities among PE mortality rates in the United States, particularly in counties with greater social vulnerability. Although our study has identified areas for further investigation, additional research using more comprehensive data is needed to validate our observations. If these disparities are confirmed, health care delivery systems should consider using the SVI to identify populations at higher risk of morbidity and mortality from PE. Thus, our study may serve as a hypothesis-generating platform for future research in this area.

## Figures and Tables

**TABLE 1 ]:** Social Vulnerability Index: Characterization of the 16 Social Factors Under Four Themes Used to Assess Social Vulnerability

Socioeconomic Status	Household Characteristics	Racial and Ethnic Minority Status	Housing Type and Transportation
Unemployed Below 150% poverty Housing cost burden No high school diploma No health insurance	Aged 17 y or younger Aged 65 y or older Single-parent households Civilian with a disability English language proficiency	Hispanic or Latino (of any race); Black and African American, Not Hispanic or Latino; American Indian and Alaska Native, Not Hispanic or Latino; Asian, Not Hispanic or Latino; Native Hawaiian and Other Pacific Islander, Not Hispanic or Latino; Two or More Races, Not Hispanic or Latino; Other Races, Not Hispanic or Latino	Group quarters Mobile homes Multi-unit structures Crowding No vehicle

**TABLE 2 ]:** Age-Adjusted Mortality Rates With Corresponding 95% CIs and Rate Differences for Pulmonary Embolism Mortality in Quartiles Based on SVI

	Cumulative	SVI-Q1	SVI-Q2	SVI-Q3	SVI-Q4	Rate Difference (SVI-Q4 – SVI-Q1)
All	2.32 (2.30–2.34)	1.97 (1.93–2.02)	2.22 (2.18–2.26)	2.51 (2.47–2.55)	2.43 (2.38–2.48)	0.46 excess deaths
Sex						
Male	2.39 (2.35–2.42)	1.98 (1.91–2.05)	2.32 (2.26–2.38)	2.57 (2.51–2.63)	2.55 (2.48–2.62)	0.57 excess deaths
Female	2.23 (2.20–2.26)	1.92 (1.86–1.99)	2.13 (2.08–2.19)	2.42 (2.37–2.48)	2.35 (2.28–2.41)	0.43 excess deaths
Race or Ethnicity						
Black	4.10 (4.01–4.19)	3.22 (2.92–3.51)	4.12 (3.89–4.34)	4.22 (4.07–4.37)	4.17 (4.02–4.33)	0.95 excess deaths
White	2.19 (2.17–2.21)	1.96 (1.91–2.01)	2.22 (2.17–2.26)	2.34 (2.30–2.39)	2.19 (2.14–2.24)	0.23 excess deaths
Asian/Pacific Islander	0.46 (0.42–0.51)	0.56 (0.42–0.72)	0.53 (0.45–0.61)	0.52 (0.42–0.61)	0.33 (0.25–0.41)	0.23 fewer deaths
American Indian/Alaska Native	1.18 (1.01–1.36)	Suppressed	1.41 (1.02–1.89)	1.33 (1.01–1.73)	1.00 (0.78–1.27)	0.41 fewer deaths^a^
Hispanic	0.98 (0.92–1.02)	0.82 (0.64–0.99)	1.09 (0.96–1.22)	0.98 (0.89–1.06)	0.97 (0.90–1.03)	0.15 excess deaths
Non-Hispanic	2.47 (2.44–2.49)	2.01 (1.96–2.06)	2.30 (2.25–2.34)	2.69 (2.64–2.73)	2.88 (2.81–2.94)	0.87 excess deaths
Geographic region						
Northeast	1.81 (1.77–1.86)	1.58 (1.50–1.66)	2.22 (2.11–2.32)	1.76 (1.67–1.84)	1.72 (1.60–1.84)	0.14 excess deaths
Midwest	2.60 (2.55–2.65)	2.21 (2.12–2.29)	2.58 (2.49–2.68)	2.84 (2.74–2.93)	3.45 (3.24–3.66)	1.24 excess deaths
South	2.88 (2.84–2.92)	2.10 (1.99–2.20)	2.45 (2.37–2.53)	2.99 (2.92–3.05)	3.34 (3.25–3.42)	1.24 excess deaths
West	1.52 (1.48–1.55)	2.09 (1.93–2.25)	1.70 (1.63–1.77)	1.60 (1.52–1.68)	1.13 (1.07–1.18)	0.96 fewer deaths
Metropolitan	2.12 (2.10–2.14)	1.93 (1.88–1.98)	2.10 (2.06–2.15)	2.30 (2.26–2.34)	1.96 (1.91–2.00)	0.03 excess deaths
Nonmetropolitan	3.41 (3.34–3.48)	2.22 (2.09–2.35)	2.90 (2.77–3.03)	3.88 (3.74–4.02)	4.12 (3.98–4.26)	1.9 excess deaths
Age						
< 35 y	0.22 (0.21–0.23)	0.17 (0.15–0.19)	0.20 (0.18–0.21)	0.22 (0.20–0.24)	0.22 (0.20–0.24)	0.05 excess deaths
35–64 y	2.16 (2.12–2.20)	1.68 (1.60–1.75)	2.09 (2.03–2.16)	2.36 (2.29–2.43)	2.38 (2.30–2.45)	0.70 excess deaths
≥ 65 y	10.90 (10.77–11.03)	9.85 (9.55–10.15)	10.43 (10.18–10.68)	11.79 (11.54–12.03)	11.13 (10.84–11.42)	1.28 excess deaths

SVI-Q indicates each quartile, a statistical measure that divides the dataset into four equal parts. SVI-Q1 is the lowest quartile and represents the 25th percentile of data; SVI-Q2 is the second quartile and represents the > 25th-5oth percentile; SVI-Q3 is the third quartile and represents the > 50th to 75th percentile; SVI-Q4 is the highest quartile and represents the > 75th to 100th percentile. Q = quartile; SVI = social vulnerability index.

a Compared SVI-Q2 and SVI-Q4.
